# Incidence of Treatment for Opioid Use Disorder Following Nonfatal Overdose in Commercially Insured Patients

**DOI:** 10.1001/jamanetworkopen.2020.5852

**Published:** 2020-05-27

**Authors:** Austin S. Kilaru, Aria Xiong, Margaret Lowenstein, Zachary F. Meisel, Jeanmarie Perrone, Utsha Khatri, Nandita Mitra, M. Kit Delgado

**Affiliations:** 1 National Clinician Scholars Program, Corporal Michael J. Crescenz Veterans Affairs Medical Center, University of Pennsylvania, Philadelphia; 2 Center for Emergency Care Policy and Research, Perelman School of Medicine, Department of Emergency Medicine, University of Pennsylvania, Philadelphia; 3 Leonard Davis Institute of Health Economics, University of Pennsylvania, Philadelphia; 4 Penn Injury Science Center, Philadelphia, Pennsylvania; 5 Perelman School of Medicine, Department of Biostatistics and Epidemiology, University of Pennsylvania, Philadelphia

## Abstract

**Importance:**

Timely initiation and referral to treatment for patients with opioid use disorder seen in the emergency department is associated with reduced mortality. It is not known how often commercially insured adults obtain follow-up treatment after nonfatal opioid overdose.

**Objective:**

To investigate the incidence of follow-up treatment following emergency department discharge after nonfatal opioid overdose and patient characteristics associated with receipt of follow-up treatment.

**Design, Setting, and Participants:**

A retrospective cohort study was conducted using an administrative claims database for a large US commercial insurer, from October 1, 2011, to September 30, 2016. Data analysis was performed from May 1, 2019, to September 26, 2019. Adult patients discharged from the emergency department after an index opioid overdose (no overdose in the preceding 90 days) were included. Patients with cancer and without continuous insurance enrollment were excluded.

**Main Outcomes and Measures:**

The primary outcome was follow-up treatment in the 90 days following overdose, defined as a combined outcome of claims for treatment encounters or medications for opioid use disorder (buprenorphine and naltrexone). Analysis was stratified by whether patients received treatment for opioid use disorder in the 90 days before the overdose. Logistic regression models were used to identify patient characteristics associated with receipt of follow-up treatment. Marginal effects were used to report the average adjusted probability and absolute risk differences (ARDs) in follow-up for different patient characteristics.

**Results:**

A total of 6451 patients were identified with nonfatal opioid overdose; the mean (SD) age was 45.0 (19.3) years, 3267 were women (50.6%), and 4676 patients (72.5%) reported their race as non-Hispanic white. A total of 1069 patients (16.6%; 95% CI, 15.7%-17.5%) obtained follow-up treatment within 90 days after the overdose. In adjusted analysis of patients who did not receive treatment before the overdose, black patients were half as likely to obtain follow-up compared with non-Hispanic white patients (ARD, −5.9%; 95% CI, −8.6% to −3.6%). Women (ARD, −1.7%; 95% CI, −3.3% to −0.5%) and Hispanic patients (ARD, −3.5%; 95% CI, −6.1% to −0.9%) were also less likely to obtain follow-up. For each additional year of age, patients were 0.2% less likely to obtain follow-up (95% CI, −0.3% to −0.1%).

**Conclusions and Relevance:**

Efforts to improve the low rate of timely follow-up treatment following opioid overdose may seek to address sex, race/ethnicity, and age disparities.

## Key Points

**Question:**

How often do commercially insured patients obtain follow-up treatment for opioid use disorder after a nonfatal opioid overdose?

**Findings:**

In this cohort study of national commercial insurance claims for 6451 patients, 16.6% of patients obtained follow-up treatment after a nonfatal opioid overdose. Among those who had not received treatment for opioid use disorder before the overdose, patients of older age, female sex, black race, and Hispanic ethnicity were less likely to obtain follow-up.

**Meaning:**

Timely treatment for opioid use disorder following overdose appears to be low among commercially insured patients, with race/ethnicity, sex, and age disparities.

## Teaser

This cohort study examines the use of follow-up treatment by patients who experience heroin or prescription opioid overdose.

## Introduction

Each year, the emergency department (ED) provides care for an increasing number of patients who present with opioid overdose as well as medical complications of opioid use disorder (OUD).^1,2,3^ The ED serves as an essential touchpoint for patients seeking care for withdrawal and addiction.^4,5,6,7^ A key strategy in secondary prevention of opioid overdose deaths is the engagement of patients with OUD in treatment following discharge.^8,9,10,11^

However, few patients successfully transition to treatment following nonfatal overdose.^12,13,14^ In evidence from 2 states, less than 5% of Medicaid patients initiated treatment with medication for opioid use disorder (MOUD) following overdose.^13,14^ For patients who are ready to engage in treatment, care coordination can help to overcome barriers to access.^4,9^ Yet hospitals have few incentives and capacity to provide resource-intensive care navigation after ED visits.^5,8,15,16,17,18^

Patients have high risk of death in the days immediately following opioid overdose.^19,20^ The initiation of MOUD during or after emergency care is associated with improvements in a variety of patient outcomes, including all-cause mortality and engagement in outpatient treatment, and other hospital-based interventions have been developed.^12,21,22,23,24^ As a consequence, policy makers have identified the transition of patients from emergency care to sustained treatment (termed *warm handoffs*) as an urgent priority.^25,26,27,28^

In this study, we sought to examine the rate of follow-up treatment after discharge from the ED following overdose in a national population of commercially insured adults. Previous studies have focused on single states, the Medicaid population, and MOUD treatment.^12,13,14,29^ To our knowledge, no previous studies have included the full scope of treatment services available to patients.

We also sought to examine patient-level characteristics associated with timely receipt of follow-up care. Evidence suggests that significant treatment disparities on the basis of race, sex, and geography have emerged as the opioid epidemic has evolved, possibly owing to differences in health insurance coverage.^30,31,32,33,34,35,36,37^ We hypothesized that these treatment disparities by race and sex would persist within a commercially insured population.

## Methods

### Data Sources, Study Population, and Outcomes

We conducted a retrospective cohort study of adult patients who were discharged from the ED following treatment for opioid overdose between October 1, 2011, and September 30, 2016. We used an administrative claims database, the Optum Clinformatics Data Mart (Optum).^38,39^ The Optum database includes all inpatient, ED, outpatient, and pharmacy claims from a large national health insurance company that enrolled between 15 million and 18 million unique patients each year during the study period. Data analysis was performed from May 1, 2019, to September 26, 2019. The institutional review board at the University of Pennsylvania determined that this study was exempt from review because data are deidentified. This study followed the Strengthening the Reporting of Observational Studies in Epidemiology (STROBE) reporting guideline for cohort studies.^40^

#### Selection of Patient Cohort

We identified ED encounters for opioid overdose in the study period for patients with commercial insurance coverage (eFigure 1 in the Supplement). To do so, we used previously validated *International Classification of Diseases, Ninth Revision, Clinical Modification* (*ICD-9-CM*) and *International Statistical Classification of Diseases, Tenth Revision, Clinical Modification* (*ICD-10-CM*) diagnosis codes before and after October 1, 2015, respectively (eTable 1 in the Supplement).^41,42,43,44^ We used Current Procedural Terminology codes to specifically identify ED encounters (eTable 1 in the Supplement).^45^

We excluded encounters for patients who did not have continuous insurance enrollment for 90 days before and after the date of the overdose, to provide a sufficient window to measure patient exposures and outcomes and exclude fatal overdoses. We excluded patients with age younger than 18 years.

We then limited the cohort to encounters for an index opioid overdose, defined as an encounter for opioid overdose with no ED encounter or hospital admission for opioid overdose in the preceding 90 days. We excluded encounters resulting in inpatient hospital admission to obtain a cohort of patients stable for ED discharge and likely to not have disability or sequelae from the overdose. In addition, we excluded encounters for patients with diagnosis of cancer based on treatment claims *ICD-9-CM* and *ICD-10* diagnosis codes in the preceding 90 days (eTable 1 in the Supplement).^12,46,47^ Patients with pain related to active cancer diagnoses represent a separate population and may be prescribed high doses of prescription opioids.^29^ Of the remaining encounters, we included only the first index opioid overdose for any individual patient during the study period (eFigure 1 in the Supplement).

#### Outcomes

The primary outcome was whether the patient obtained follow-up treatment in the 90 days following the index opioid overdose. We defined follow-up treatment as the presence of either 1 pharmacy claim for MOUD or 1 medical claim for an outpatient or inpatient opioid treatment encounter. For pharmacy claims, we identified National Drug Codes for all formulations of buprenorphine, buprenorphine with naloxone, or naltrexone (eTable 2 in the Supplement).^48,49,50^ Methadone maintenance therapy was not covered by insurance for this population during the study period and was not included in this study. Medical claims for treatment encounters had an *ICD-9-CM* or *ICD-10-CM* diagnosis code for opioid use disorder in any position (eTable 3 in the Supplement) and Current Procedural Terminology or Healthcare Common Procedure Coding System codes for a variety of services including outpatient clinic visits, psychiatric services, inpatient and outpatient behavioral health services, outpatient treatment programs, and case management (eTable 3 in the Supplement).^50^ Repeated ED or inpatient hospital encounters were not included as follow-up treatment.

Supplemental analyses were performed for the purpose of hypothesis generation. These included secondary outcomes that were the receipt of MOUD independently from treatment encounters within 90 days of the index overdose. We also examined the number of days from the index overdose to follow-up treatment. To address the absence of mortality data, we determined the date of service for the last insurance claim for all patients in the cohort. We performed a sensitivity analysis excluding patients for whom there was no claim beyond the 90-day follow-up period. Although the absence of claims does not indicate death, we could not ensure survival to the end of the follow-up period for those patients.

#### Covariates

We examined patient-level characteristics as covariates that we hypothesized could be associated with access to follow-up treatment, including patient age, sex, and race/ethnicity. Optum uses data on race/ethnicity that is self-reported or derived from administrative data sources. We also included geographic location, according to 4 United States Census Regions (Northeast, South, Midwest, West).^51^ Year of the index overdose was included given the increasing overdose incidence over the study period.^52^ We examined the type of overdose (heroin or prescription opioid) based on diagnosis codes.^41^ Prescription opioid refers to medications available by prescription but does not mean that the patient received a prescription for the medication.

We also included exposures to treatment for behavioral health conditions in the 90 days preceding the index overdose. We included the presence of claims for anxiety or depression based on *ICD-9-CM* or *ICD-10-CM* diagnosis codes (eTable 1 in the Supplement) due to potential association with overdose.^50^ We also included claims for prescription opioid medications and benzodiazepines in the 90 days preceding the index overdose using American Hospital Formulary Service Pharmacologic-Therapeutic Classification codes.^53^ In addition, we determined whether patients had pharmacy claims for MOUD or medical claims for treatment encounters in the 90 days preceding the index overdose.

### Statistical Analysis

First, we described the patient cohort, stratified by overdose type. We used 2-sided χ^2^ tests and *t* tests to describe differences in the cohort between overdose type. Next, we summarized patient outcomes, stratified by overdose type and treatment for OUD in the 90 days preceding the overdose.

We then used multivariable logistic regression models to examine the association between patient characteristics, as described in the first paragraph of the Covariates section, and the binary primary outcome. Given that patients were hypothesized to more likely access follow-up treatment if they had received recent treatment before the overdose, we stratified the analyses based on whether patients had received OUD treatment in the 90 days before the overdose. For ease of interpretation, we used predictive margins to report average adjusted probability and absolute risk differences (ARDs), with 95% CIs.^54,55^ For categorical variables, ARD represents the difference in adjusted probability of follow-up treatment between patients with a given characteristic and the reference value.

In addition to the primary analysis, we investigated potential interactions between race/ethnicity and overdose type by including an interaction term in the logistic regression model. Also, we used multivariable logistic regression models to examine the association between patient characteristics and the secondary outcome of MOUD treatment alone. In addition, we used Kaplan-Meier failure analysis to examine days to receipt of follow-up treatment, stratified by overdose type. Data analysis was conducted from June 1, 2019, to September 1, 2019. Analyses were performed using Stata software, version 15.1 (StataCorp LP).

## Results

The total cohort consisted of 6451 patients, of whom 1896 (29.4%) overdosed from heroin and 4555 (70.6%) overdosed from prescription opioids (Table 1). Further delineation of the type of opioid overdose is reported in eTable 7 in the Supplement. The mean (SD) age was 45.0 (19.3) years and there were 3267 (50.6%) women. A total of 4676 patients (72.5%) reported their race as non-Hispanic white, 601 patients (9.3%) reported their race as black, and 536 patients (8.3%) who reported Hispanic ethnicity. Only 682 patients (10.6%) received treatment for opioid use disorder in the 90 days preceding the overdose, including 320 (5.0%) with pharmacy claims for MOUD. Patients with heroin overdose significantly differed across all patient characteristics compared with those with prescription opioid overdose.

**Table 1.** Characteristics of Patient Cohort, Stratified by Overdose Type^a^

Characteristic	No. (%)		
	All patients (n = 6451)	Overdose	
		Heroin (n = 1896)	Prescription opioid (n = 4555)
Age, mean (SD), y	45.0 (19.3)	31.0 (13.2)	50.8 (18.4)
Sex			
Male	3184 (49.4)	1291 (68.1)	1893 (41.6)
Female	3267 (50.6)	605 (31.9)	2662 (58.4)
Race/ethnicity			
Non-Hispanic white	4676 (72.5)	1450 (76.5)	3226 (70.8)
Black	601 (9.3)	148 (7.8)	453 (9.9)
Hispanic	536 (8.3)	135 (7.1)	401 (8.8)
Asian	78 (1.2)	9 (0.5)	69 (1.5)
Unknown	560 (8.7)	154 (8.1)	406 (8.9)
Year			
2011, quarter 4	229 (3.5)	40 (2.1)	189 (4.1)
2012	1099 (17.1)	239 (12.6)	860 (18.9)
2013	1164 (18.1)	276 (14.6)	888 (19.5)
2014	1248 (19.3)	362 (19.1)	886 (19.5)
2015	1387 (21.5)	475 (25.1)	912 (20.0)
2016, quarters 1-3	1324 (20.5)	504 (26.6)	820 (18.0)
Region			
Northeast	659 (10.2)	316 (16.7)	343 (7.5)
South	2627 (40.7)	617 (32.5)	2010 (44.1)
Midwest	1619 (25.1)	703 (37.1)	916 (20.1)
West	1546 (24.0)	260 (13.7)	1286 (28.2)
90 d Before overdose			
Anxiety treatment	1625 (25.2)	403 (21.3)	1222 (26.8)
Depression treatment	1416 (22.0)	322 (17.0)	1094 (24.0)
Prescription opioid claim	3266 (50.6)	317 (16.7)	2949 (64.7)
Benzodiazepine claim	2009 (31.1)	373 (19.7)	1636 (35.9)
MOUD claim	320 (5.0)	201 (10.6)	119 (2.6)
Buprenorphine	278 (4.3)	168 (8.9)	110 (2.4)
Naltrexone	42 (0.7)	33 (1.7)	9 (0.2)
Treatment encounter for OUD	539 (8.4)	347 (18.3)	192 (4.2)

Abbreviations: MOUD, medication for opioid use disorder; OUD, opioid use disorder.

^a^ Two-sided *t* test and χ^2^ tests were performed; *P* < .001 for all patient characteristics.

### Primary Analysis

**Figure 1.**
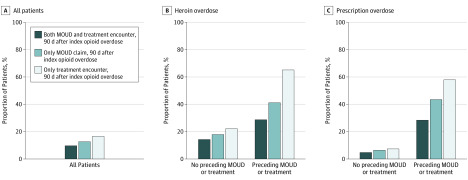
Patient Outcomes Stratified by Overdose Type and Treatment Status Before Overdose Data shown for status at 90 days before overdose for all patients (A), heroin overdose (B), and prescription opioid overdose (C). MOUD indicates medication for opioid use disorder.

For all patients in the study cohort, 1069 individuals (16.6%; 95% CI, 15.7%-17.5%) obtained follow-up treatment in the 90 days following overdose (Figure 1; eTable 8 in the Supplement). Among the 5769 patients who did not receive treatment for OUD in the 90 days before the overdose, 643 (11.1%; 95% CI, 10.3%-12.0%) obtained follow-up treatment. Among the 682 patients who received treatment before the overdose, 426 individuals (62.5%; 95% CI, 58.7%-66.1%) patients obtained follow-up.

**Figure 2.**
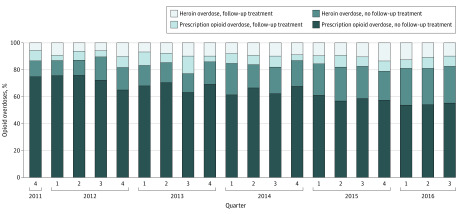
Proportion of Index Opioid Overdoses by Quarter, Stratified by Overdose Type and Receipt of Follow-up Treatment

In the adjusted analysis for patients who did not receive treatment before the overdose, patients with prescription opioid overdose were less likely to obtain follow-up compared with heroin overdose (Table 2) (ARD, −8.8%; 95% CI, −11.2% to −6.5%). Compared with patients of non-Hispanic white race, black (ARD, −5.9%; 95% CI, −8.6% to −3.6%) and Hispanic (ARD, −3.5%; 95% CI, −6.1% to −0.9%) patients were less likely to obtain follow-up. Women were less likely to obtain follow-up than men (ARD, −1.7%; 95% CI, −3.3% to −0.5%). For each additional year of age, patients were 0.2% less likely to obtain follow-up (95% CI, −0.3% to −0.1%). However, patients with recent treatment for anxiety, including a treatment encounter for anxiety (ARD, 3.4%, 95% CI, 1.1%-5.8%) or prescription for a benzodiazepine (ARD, 2.8%; 95% CI, 0.7%-5.0%), were more likely to obtain follow-up. In this adjusted analysis, there was no statistically significant change with regard to the rate of patients obtaining follow-up treatment over the 5 years of the study (Figure 2).

**Table 2.** Adjusted Probability of Follow-up Treatment After Opioid Overdose, for Patients Not Treated Before Overdose^a^

Patient characteristics	Average adjusted probability, % (95% CI)^b^	*P* value^c^
Overdose type		
Prescription opioid	8.3 (7.3- 9.2)	[Reference]
Heroin	17.1 (15.1-19.2)	<.001
Age, at mean, y^d^	9.9 (9.1-10.7)	<.001
Sex		
Male	11.9 (10.9-13.0)	[Reference]
Female	10.1 (9.1-11.3)	.04
Race/ethnicity		
Non-Hispanic white	12.1 (11.1-13.0)	[Reference]
Black	6.1 (4.0-8.3)	<.001
Hispanic	8.5 (6.1-11.0)	.009
Asian	10.2 (2.8-17.5)	.62
Unknown	10.1 (7.4-12.8)	.18
Year		
2011, quarter 4	12.2 (7.9-16.6)	[Reference]
2012	9.3 (7.6-11.3)	.22
2013	11.5 (9.6-13.5)	.75
2014	10.0 (8.3-11.7)	.32
2015	12.9 (11.1-14.6)	.82
2016, quarters 1-3	11.1 (9.5-13.0)	.64
Region		
Northeast	14.0 (11.6-16.6)	[Reference]
South	10.4 (9.1-11.4)	.01
Midwest	11.1 (9.7-12.7)	.07
West	11.0 (9.3-12.8)	.06
90 d Before overdose		
Anxiety treatment		
No	10.3 (9.4-11.2)	[Reference]
Yes	13.8 (11.7-15.8)	.004
Depression treatment		
No	10.9 (10.1-11.9)	[Reference]
Yes	11.6 (9.7-13.5)	.64
Prescription opioid claim		
No	11.0 (9.9-12.1)	[Reference]
Yes	11.2 (9.8-12.7)	.84
Benzodiazepine claim		
No	10.3 (9.4-11.2)	[Reference]
Yes	13.2 (11.4-15.0)	.009

^a^ Results are given for patients who did not receive treatment for 90 days before the index opioid overdose, defined as either a pharmacy claim for medication for opioid use disorder or medical claim for opioid use disorder treatment encounter.

^b^ Estimated with logistic regression model using predictive margins. Average adjusted probability is the adjusted rate, holding covariates at their actual values, at which patients obtain follow-up treatment within 90 days after the index opioid overdose, defined as either a pharmacy claim for medication for opioid use disorder or medical claim for opioid use disorder treatment encounter.

^c^ *P* values are given for average marginal effects, which represent the difference in adjusted probability between a given characteristic and the reference group.

^d^ Average adjusted probability for continuous variable (age) is given for the mean patient age (46.3 years).

These associations were not present for patients who received treatment in the 90 days before overdose, apart from a decreased rate of follow-up for patients in the South (ARD, −15.0%; 95% CI, −25.9% to −4.1% and the West (ARD, −20.1%; 95% CI, −32.% to −7.6%), compared with the Northeast (eTable 4 in the Supplement).

### Supplemental Analyses

**Figure 3.**
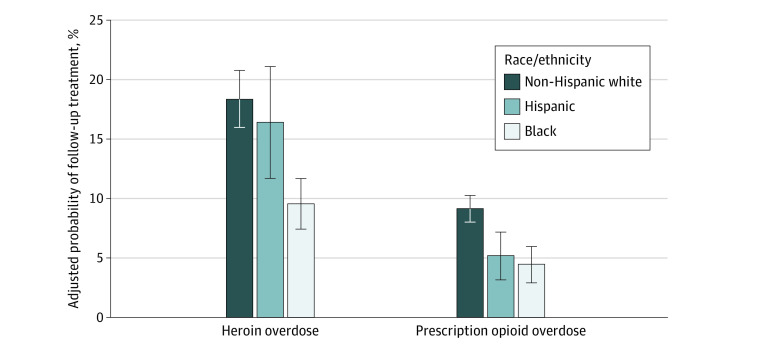
Average Adjusted Probability of Follow-up Treatment After Opioid Overdose, by Overdose Type and Race/Ethnicity Estimated from logistic regression model with interaction term for overdose type and race/ethnicity. Error bars denote 95% confidence intervals for average adjusted probability. Results shown only for patients who had not received treatment for opioid use disorder in the 90 days before the index opioid overdose. Race/ethnicity was self-reported or derived from other administrative data sources.

In supplemental analyses, differences in the adjusted probability of follow-up rate persisted across overdose type for black patients compared with non-Hispanic white patients (Figure 3). Among patients who did not receive treatment before overdose, black patients were less likely to obtain follow-up treatment than non-Hispanic white patients whether the index overdose was due to heroin (ARD, −8.8%; 95% CI, −11.5% to −6.1%) or prescription opioids (ARD, −4.7%; 95% CI, −5.7% to −3.7%). For Hispanic patients compared with patients of non-Hispanic white race, the difference in adjusted follow-up rate was significant only for patients with prescription opioid overdose (ARD, −4.0%; 95% CI, −5.% to 2.8%).

We investigated the secondary outcome of MOUD treatment alone. Among the 6131 patients who did not file an MOUD claim in the 90 days before the index overdose, 280 individuals (4.6%) had a claim for MOUD following the overdose. In adjusted analyses, patients who were older, women, black race, and experienced a prescription opioid overdose were less likely to obtain MOUD treatment, while patients with a prescription for a benzodiazepine or treatment encounters for OUD were more likely (eTable 5 in the Supplement).

We examined the timing of follow-up treatment following the index overdose, with results of the Kaplan-Meier failure analysis shown in eFigure 2 in the Supplement. Among all 1069 patients who obtained follow-up treatment, 318 individuals (29.7%) did so in 7 or fewer days after the overdose. In addition, we performed a sensitivity analysis excluding 233 patients (3.6%) who did not have claims beyond the 90-day follow-up period, which demonstrated equivalent outcomes to the primary analysis (eTable 6 in the Supplement).

## Discussion

We analyzed commercial insurance claims to determine how often patients obtained treatment for OUD in the 90 days following ED presentation for nonfatal opioid overdose. Most had not received OUD treatment immediately preceding the overdose. Among that group, we found that only 11.1% of patients obtained follow-up treatment through an encounter in the outpatient setting, inpatient treatment, or filled prescriptions for a buprenorphine or naltrexone. The few patients that recently received treatment had a higher incidence of follow-up treatment. Despite the increasing number of overdoses across the years of this study, there was no significant change in the proportion of patients receiving follow-up treatment. Given that patients with commercial insurance likely have a superior ability to access care compared with patients who have public insurance, this persistently low rate suggests an opportunity for improvement.

Disparities in the receipt of follow-up treatment with regard to race/ethnicity, age, and age persisted within this cohort. In particular, black patients were half as likely to obtain treatment following overdose compared with non-Hispanic white patients. This disparity was present regardless of whether the overdose was due to heroin or prescription opioids. To our knowledge, these disparities in treatment following opioid overdose have not been previously documented. However, our findings are consistent with emerging evidence that there are disparities in buprenorphine treatment with regard to race/ethnicity and sex.^30,32,33,36^ Although this study cannot determine whether these disparities are associated with patient preferences, barriers to access, implicit or explicit bias, or other causes, it is important to better understand and account for these factors when designing systems that seek to improve engagement and equity in treatment.

Previous studies have examined changes in treatment rates before and after opioid overdose using data from individual states.^12,13,14^ These studies primarily focused on medication treatment, with only one study including a limited range of treatment encounters. Our study included a range of possible treatments, from outpatient clinic visits to inpatient residential treatment. In general, we found that fewer than half of patients who obtained follow-up treatment received medication. Treatment with opioid agonists has been associated with reduced risk of relapse by 50% compared with behavioral treatment alone.^56^ Better understanding of current treatment and referral patterns may help inform efforts to expand evidence-based practices.^57,58^

We hypothesized that the rate of follow-up treatment would be higher for patients with commercial insurance, given potentially greater resources and access to care. While we cannot directly compare across studies, the rate of OUD treatment in this cohort did not appear to be appreciably higher in this cohort than that described in other populations. Not all patients can be expected to engage in treatment after overdose.^4^ Higher rates of treatment engagement have been observed in experimental settings, often with screening of patients for substance use disorder.^22,59,60,61,62^ While the optimal rate of follow-up treatment may be difficult to estimate, there is still need for widely implemented interventions that may help patients overcome the many pervasive barriers to accessing care.^4,15^

We intentionally examined outcomes for a short time following the overdose. Recent evidence suggests that risk of death is high immediately following overdose, with nearly 5% of deaths occurring within 2 days of discharge from the ED.^19^ In a secondary analysis, only 30% of patients who obtained follow-up did so within 7 days. Patients may benefit from rapid linkage to treatment, potentially through recovery specialists who can provide navigation and harm reduction counseling regardless of the client’s willingness to engage in treatment.^3,4,5^

### Limitations

This study has several limitations. First, we cannot account for patients who pay for OUD treatment out-of-pocket. Although treatment services, including MOUD, were covered by the insurer during the study period, some patients may have elected to pursue alternative options. Second, this study did not include patients who obtain methadone maintenance therapy. Methadone is an important treatment modality for many patients with opioid use disorder. However, methadone was not covered for this indication by the insurer during the study period. It is possible that patients in this cohort obtained methadone through self-pay or other mechanisms, although this rate cannot be estimated from these data and is difficult to extrapolate from other sources.^63,64,65^ Third, these data do not specifically account for patient deaths in the days following the index overdose. However, additional analysis that only included patients known to have survived to the end of the follow-up period showed similar results.

Fourth, the use of administrative claims data in this study limits our ability to ascertain the reasons that patients obtain or do not obtain follow-up treatment. It is not known whether patients do not receive appropriate referrals, lack treatment facilities in their communities, or may be unwilling to engage in treatment. A corollary limitation is that patients may have received prescriptions for MOUD but not filled those prescriptions. Fifth, this cohort likely includes patients who may not have OUD, which may explain differential rates in follow-up treatment for patients with heroin and prescription opioid overdose. Regardless, patients with accidental prescription opioid overdose also should obtain timely follow-up for reevaluation, medication adjustment, and discussion of the long-term risks associated with opioid use.

## Conclusions

Engagement of patients into treatment following opioid overdose is necessary to prevent subsequent opioid overdose death and other harm. Among commercially insured patients who were not receiving active addiction treatment, only 11.1% received follow-up treatment after an overdose. We showed apparent disparities in treatment with regard to race/ethnicity (eg, black patients were half as likely to obtain follow-up compared with non-Hispanic white patients), sex, and age. Research is needed to better understand the mechanisms behind these disparities. As health professionals adopt evidence-based practices for initiating medications for treatment of OUD and linking patients to sustained treatment, payers and policy makers should implement strategies to overcome systemic barriers to ensure that patients are given the best opportunity to access timely treatment. These interventions must account for disparities to ensure expanded and equitable access to life-saving treatment following overdose.

## Notes

Supplement. **eFigure 1.** Flowchart for Selection of Patient Cohort **eTable 1.** *ICD-9-CM*, *ICD-10*, CPT, and AHFS Codes for Selection of Patient Cohort and Patient Characteristics **eTable 2.** National Drug Codes for Medications for Opioid Use Disorder **eTable 3.** CPT, HCPCS, *ICD-9-CM*, and *ICD-10-CM* Codes for Treatment Encounters **eTable 4.** Adjusted Probability of Follow-up Treatment After Opioid Overdose for Patients Treated Prior to Overdose **eTable 5.** Adjusted Probability of MOUD Treatment After Opioid Overdose, Stratified by Treatment Status Prior to Overdose **eFigure 2.** Kaplan-Meier Failure Curve for Days to First Follow up Treatment Following Index ED Overdose **eTable 6.** Adjusted Probability of Follow-up Treatment After Opioid Overdose, Excluding Patients Without Known Claims Beyond 90-Day Follow-up Period (Sensitivity Analysis to Address Potential Mortality During Follow-up Period) **eTable 7.** Index Opioid Overdoses by specific *ICD-9* or *ICD-10* Diagnosis Code, With Number and Frequency for Each Diagnosis Code **eTable 8.** Patient Cohort and Unadjusted Outcomes, Stratified by Overdose Type and Treatment Status Before Overdose
