# Supplementary material for: Incidence of Treatment for Opioid Use Disorder Following Nonfatal Overdose in Commercially Insured Patients
Source: JAMA Netw Open. 2020 May 27;3(5):e205852. doi: 10.1001/jamanetworkopen.2020.5852 (PMC7254182; doi:10.1001/jamanetworkopen.2020.5852)
Supplement: Supplement. — eFigure 1. Flowchart for Selection of Patient Cohort eTable 1. ICD-9-CM, ICD-10, CPT, and AHFS Codes for Selection of Patient Cohort and Patient Characteristics eTable 2. National Drug Codes for Medications for Opioid Use Disorder eTable 3. CPT, HCPCS, ICD-9-CM, and ICD-10-CM Codes for Treatment Encounters eTable 4. Adjusted Probability of Follow-up Treatment After Opioid Overdose for Patients Treated Prior to Overdose eTable 5. Adjusted Probability of MOUD Treatment After Opioid Overdose, Stratified by Treatment Status Prior to Overdose eFigure 2. Kaplan-Meier Failure Curve for Days to First Follow up Treatment Following Index ED Overdose eTable 6. Adjusted Probability of Follow-up Treatment After Opioid Overdose, Excluding Patients Without Known Claims Beyond 90-Day Follow-up Period (Sensitivity Analysis to Address Potential Mortality During Follow-up Period) eTable 7. Index Opioid Overdoses by specific ICD-9 or ICD-10 Diagnosis Code, With Number and Frequency for Each Diagnosis Code eTable 8. Patient Cohort and Unadjusted Outcomes, Stratified by Overdose Type and Treatment Status Before Overdose [file jamanetwopen-3-e205852-s001.pdf]

Supplementary Online Content

Kilaru AS, Xiong A, Lowenstein M, et al. Incidence of treatment for opioid use disorder following nonfatal overdose in commercially insured patients. *JAMA Netw Open*. 2020;3(5):e205852. doi:10.1001/jamanetworkopen.2020.5852

eFigure 1. Flowchart for Selection of Patient Cohort

eTable 1. *ICD-9-CM*, *ICD-10*, CPT, and AHFS Codes for Selection of Patient Cohort and Patient Characteristics

eTable 2. National Drug Codes for Medications for Opioid Use Disorder

eTable 3. CPT, HCPCS, *ICD-9-CM*, and *ICD-10-CM* Codes for Treatment Encounters

eTable 4. Adjusted Probability of Follow-up Treatment After Opioid Overdose for Patients Treated Prior to Overdose

eTable 5. Adjusted Probability of MOUD Treatment After Opioid Overdose, Stratified by Treatment Status Prior to Overdose

eFigure 2. Kaplan-Meier Failure Curve for Days to First Follow up Treatment Following Index ED Overdose

eTable 6. Adjusted Probability of Follow-up Treatment After Opioid Overdose, Excluding Patients Without Known Claims Beyond 90-Day Follow-up Period (Sensitivity Analysis to Address Potential Mortality During Follow-up Period)

eTable 7. Index Opioid Overdoses by specific *ICD-9* or *ICD-10* Diagnosis Code, With Number and Frequency for Each Diagnosis Code

eTable 8. Patient Cohort and Unadjusted Outcomes, Stratified by Overdose Type and Treatment Status Before Overdose

This supplementary material has been provided by the authors to give readers additional information about their work.

© 2020 Kilaru AS et al. *JAMA Network Open.*

eFigure 1. Flowchart for Selection of Patient Cohort

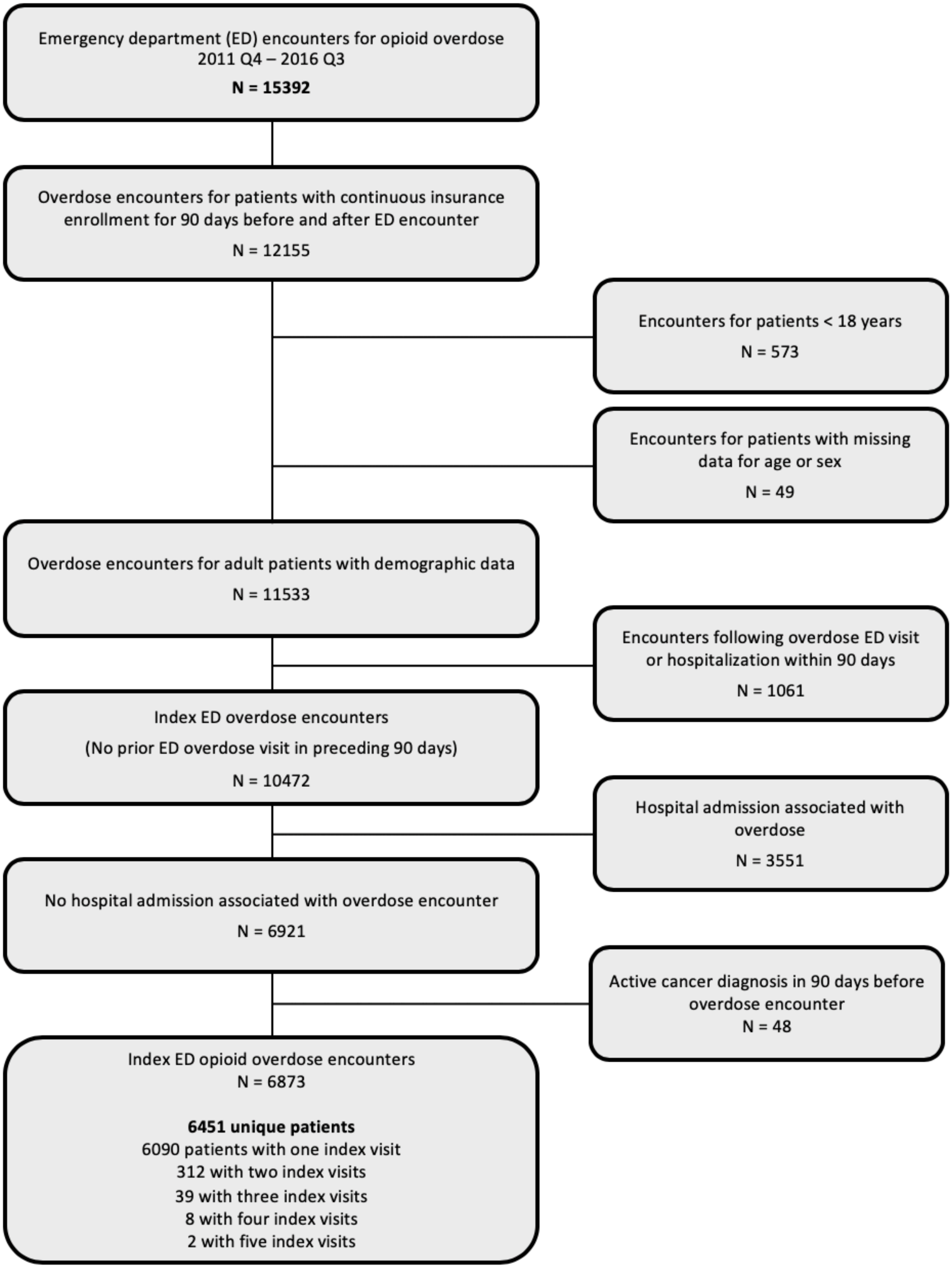

© 2020 Kilaru AS et al. *JAMA Network Open.*

eTable 1 *ICD-9-CM*, *ICD-10*, CPT, and AHFS Codes for Selection of Patient Cohort and Patient Characteristics

	ICD-9-CM [a] Diagnosis Codes	ICD-10 [a] Diagnosis Codes	CPT Codes	AHFS Pharmacologic-Therapeutic Codes
**Opioid Overdose**	Heroin:<br>965.01, E850.0<br><br>Prescription:<br>965.00, 965.02, 965.09, E.850.1, E.850.2	Heroin:<br>T40.1X<br><br>Prescription:<br>T40.0X, T40.2-4X, T40.6X		
**Emergency Department Encounters**			99281, 99282, 99283, 99284, 99285	
**Cancer (Malignant Neoplasm)[b] Diagnosis**	140.X – 208.X<br>209.0 – 209.3<br>V10.X	C00X – C97X		
**Anxiety Diagnosis**	293.84, 300.00, 300.01, 300.02, 300.09, 300.10, 300.20, 300.21, 300.22, 300.23, 300.29, 300.3, 300.5, 300.89, 300.9, 308.0, 308.1, 308.2, 308.3, 308.4, 308.9, 309.81, 313.0, 313.1, 313.21, 313.22, 313.3, 313.82, 313.83	F06.4, F40.00, F40.01, F40.02, F40.10, F40.11, F40.210, F40.218, F40.220, F40.228, F40.230, F40.231, F40.232, F40.233, F40.240, F40.241, F40.242, F40.243, F40.248, F40.290, F40.291, F40.298, F40.8, F40.9, F41.0, F41.1, F41.3, F41.8, F41.9, F42, F42.2, F42.3, F42.4, F42.8, F42.9, F43.0, F43.10, F43.11, F43.12, F44.9, F45.8, F48.8, F48.9, F93.8, F99, R45.2, R45.5, R45.6, R45.7		
**Depression Diagnosis**	296.20, 296.21, 296.22, 296.23, 296.24, 296.25, 296.26, 296.30, 296.31, 296.32, 296.33, 296.34, 296.35, 296.36, 300.4, 311, V79.0	F32.0, F32.1, F32.2, F32.3, F32.4, F32.5, F32.89, F32.9, F33.0, F33.1, F33.2, F33.3, F33.8, F33.40, F33.41, F33.42, F33.9, F34.1		
**Prescription Opioid Full Agonist**				280808
**Benzodiazepine**				281208, 282408

© 2020 Kilaru AS et al. *JAMA Network Open.*

[a] ICD-9-CM diagnosis codes are used for any claim prior to October 1 2015. ICD-10 diagnosis codes are used for claims on that date or after.
[b] Benign neoplasms or neoplasms of uncertain behavior were excluded

© 2020 Kilaru AS et al. *JAMA Network Open.*

eTable 2. National Drug Codes for Medications for Opioid Use Disorder

	National Drug Codes (NDC)
**Buprenorphine and Buprenorphine-Naloxone**	54017613, 54017713, 54018813, 54018913, 74201201, 74201232, 93360021, 93360040, 93360121, 93360140, 93360221, 93360240, 93360321, 93360340, 93537856, 93537956, 93572056, 93572156, 149075701, 228315303, 228315403, 228315473, 228315503, 228315567, 228315573, 228315603, 378092393, 378092493, 406192303, 406192403, 406802003, 409201203, 409201232, 490005100, 490005130, 490005160, 490005190, 12496010001, 12496010002, 12496010005, 12496030001, 12496030002, 12496030005, 12496075701, 12496075705, 12496120201, 12496120203, 12496120401, 12496120403, 12496120801, 12496120803, 12496121201, 12496121203, 12496127802, 12496128302, 12496130602, 12496131002, 16590066605, 16590066630, 16590066705, 16590066730, 16590066790, 21695051510, 23490927003, 23490927006, 23490927009, 35356000407, 35356000430, 35356055530, 35356055630, 35356060504, 35356060704, 38779088800, 38779088801, 38779088803, 38779088805, 38779088806, 38779088809, 40042001001, 42023017901, 42023017905, 42291017430, 42291017530, 42858035340, 42858049340, 42858050103, 42858050203, 42858058640, 42858075040, 42858083940, 43063018407, 43063018430, 43063066706, 43063075306, 49452129201, 49452129202, 49452129203, 49452825301, 49452825302, 49452825303, 49999039507, 49999039515, 49999039530, 49999063830, 49999063930, 50268014411, 50268014415, 50268014511, 50268014515, 50383028793, 50383029493, 50383092493, 50383093093, 51552076501, 51552076502, 51552076505, 51552076506, 51552076509, 51552076510, 51552076550, 51927101200, 52959030430, 52959074930, 53217013830, 54123011430, 54123090730, 54123091430, 54123092930, 54123095730, 54123098630, 54569141600, 54569141601, 54569549600, 54569573900, 54569573901, 54569573902, 54569632500, 54569632600, 54569639900, 54569640800, 54569657800, 54868570700, 54868570701, 54868570702, 54868570703, 54868570704, 54868575000, 55045378403, 55390010010, 55700014730, 55700018430, 55700030230, 55700030330, 55700057904, 58284010014, 59011075004, 59011075104, 59011075204, 59011075704, 59011075804, 59385001201, 59385001230, 59385001401, 59385001430, 59385001601, 59385001630, 59385002101, 59385002160, 59385002201, 59385002260, 59385002301, 59385002360, 59385002401, 59385002460, 59385002501, 59385002560, 59385002601, 59385002660, 59385002701, 59385002760, 60429058611, 60429058630, 60429058633, 60429058711, 60429058730, 60429058733, 62756045983, 62756046083, 62756096983, 62756097083, 62991158301, 62991158302, 62991158303, 62991158304, 62991158306, 62991158307, 62991158308, 63275992201, 63275992202, 63275992203, 63275992204, 63275992205, 63275992207, 63370090506, 63370090509, 63370090510, 63370090515, 63459030042, 63481016101, 63481016160, 63481020701, 63481020760, 63481034801, 63481034860, 63481051901, 63481051960, 63481068501, 63481068560, 63481082001, 63481082060, 63481095201, 63481095260, 63629402801, 63629403401, 63629403402, 63629403403, 63629409201, 63874108403, 63874108503, 63874117303, 65162041503, 65162041603, 65757030001, 65757030202, 66336001630, 68071138003, 68071151003, 68258299103, 68258299903, 68308020230, 68308020830,<br>35356060604, 53217024630, 55887031204, 55887031215, 63874117403, 66336001530, 406192309, 406192409, 406800503, 50090157100, 55700056804, 60846097003, 60846097103, 62175045232, 62175045832, 62756045964, 62756046064, 62756096964, 62756097064, 63629409202, 63629507401, 63629712501, 63629712502, 63629712503, 63629712504, 63629712505, 63629712506, 63629712507, 63629712601, 63629712602, 63629712603, 63629712604, 63629712605, 63629712606, 63629712607, 63629712608, 63629727001, 63629727002, 64725093003, 64725093004, 64725192403, 64725192404, 65162041509, 65162041609, 71335035301, 71335035302, 71335035303, 71335035304, 71335035305, 71335035306, 71335035307, 76519117000, 76519117001, 76519117002, 76519117003, 76519117004

© 2020 Kilaru AS et al. *JAMA Network Open.*

**Naltrexone**	43063059115, 47335032683, 47335032688, 50436010501, 51224020630, 51224020650, 51285027501, 51285027502, 52152010502, 52152010504, 52152010530, 54868557400, 63459030042, 65694010003, 65694010010, 65757030001, 65757030202, 68084029111, 68084029121, 68094085362, 68115068030, 56001122, 56001130, 56001170, 56007950, 56008050, 185003901, 185003930, 406009201, 406009203, 406117001, 406117003, 555090201, 555090202, 16729008101, 16729008110, 42291063230

© 2020 Kilaru AS et al. *JAMA Network Open.*

eTable 3 CPT, HCPCS, ICD-9-CM, and ICD-10-CM Codes for Treatment Encounters

**Current Procedure Terminology (CPT) Codes**	**Healthcare Common Procedure Coding System (HCPCS) Codes**	**ICD-9-CM Diagnosis Codes** (claim prior to October 1 2015)	**ICD-10-CM Diagnosis Codes** (claim on or after October 1 2015)
Office or Outpatient Visit:<br>99201-99205<br>99211-99215<br><br>Psychiatric Diagnosis:<br>90791-90792<br><br>Psychotherapy Services:<br>90832-90839<br>90853<br>90863<br>90875-90876<br>90801-90815<br>90824<br>90862<br><br>Screening, Brief Intervention, and Referral to Treatment (SBIRT):<br>99408-99409	Drug, Alcohol, and Behavioral Health Services (Outpatient and Inpatient):<br>H0001, H0002<br>H0004-H0019<br>H0031-H0040<br>G0396-G0397<br><br>Mental Health Services NOS:<br>H0046-H0047<br><br>Halfway House / Treatment Program:<br>H2034-H2036<br><br>Clinic Visit / Case Management:<br>T1015-T1017<br>T1001<br><br>MOUD Codes:<br>J0571-J0575<br>J1230, J2315<br><br>**Excluded**<br>*Methadone maintenance therapy:*<br>*H0020*	304.00-304.03<br>305.50-305.53<br>304.70-304.73<br>965.00-965.02, 965.09<br>E85.00-E85.02<br>E93.50-E93.51	Opioid use, abuse, dependence:<br>F1190, F11920-F11922, F11929<br>F1193, F1194, F11950-F11951, F11959, F11981, F11982, F11988<br>F1199, F1110, F11120-F11122,<br>F1129, F1114, F11150-F11151, F11159, F11181, F11182, F11188, F1119, F1120, F11220-F11222, F11229, F1123, F1124, F11250, F11251, F11259, F11281, F11282, F11288, F1129<br><br>Poisoning:<br>T400X1*, T400X2*, T400X3*,T400X4*<br>T401X1*, T401X2*, T401X3*, T401X4*<br>T403X1*, T403X2*, T403X3*, T403X4*<br>T402X1*, T402X2*, T402X3*, T402X4*<br>T404X1*, T404X2*, T404X3*, T404X4*

© 2020 Kilaru AS et al. *JAMA Network Open.*

			T40601*, T40602*, T40603*, T40604*<br>T40605*, T40691*, T40692*, T40693*, T40694*, T40695*, T403X5*<br><br>* Position can include A, D, or S

© 2020 Kilaru AS et al. *JAMA Network Open.*

eTable 4 Adjusted probability of follow-up treatment after opioid overdose for patients treated prior to overdose

		**Average Adjusted Prediction** (95% CI), %	***P***
**Overdose Type**			
	Prescription	64.2 (57.5 to 70.7)	--
	Heroin	61.3 (56.0 to 66.5)	.55
**Age (years),** mean (SD)		62.5 (59.0 to 66.1)	.21
**Sex**			
	Male	62.2 (57.5 to 70.0)	--
	Female	62.7 (57.4 to 68.2)	.89
**Race/Ethnicity**			
	White	62.8 (58.7 to 66.9)	--
	Black	75.9 (63.4 to 88.4)	.06
	Hispanic	61.3 (47.3 to 75.3)	.84
	Asian	59.6 (13.8 to 105.3)	.89
	Unknown	51.7 (39.1 to 64.1)	.10
**Year**			
	2011 Q4	48.3 (22.2 to 74.5)	--
	2012	66.0 (56.1 to 75.8)	.22
	2013	66.9 (58.9 to 75.8)	.19
	2014	60.9 (52.8 to 69.0)	.37
	2015	54.8 (46.9 to 62.7)	.65
	2016 Q1-3	66.4 (59.2 to 73.6)	.19
**Region**			
	Northeast	73.3 (64.7 to 82.0)	
	South	58.3 (51.9 to 64.1)	.007
	Midwest	67.3 (61.0 to 73.6)	.26
	West	53.2 (44.4 to 62.0)	.002
**Anxiety treatment,** 90 d prior to overdose	No	62.3 (57.6 to 67.1)	
	Yes	62.6 (56.8 to 68.5)	.94
**Depression treatment,** 90 d prior to overdose	No	63.3 (59.0 to 67.6)	
	Yes	60.7 (54.1 to 67.2)	.53
**Prescription opioid claim,** 90 d prior to overdose	No	65.1 (60.4 to 69.8)	
	Yes	56.9 (48.9 to 64.8)	.11
**Benzodiazepine claim**, 90 d prior to overdose	No	60.1 (55.4 to 64.7)	
	Yes	66.7 (60.7 to 72.6)	.10

© 2020 Kilaru AS et al. *JAMA Network Open.*

eTable 5 Adjusted probability of MOUD treatment after opioid overdose, stratified by treatment status prior to overdose

		No MOUD in 90 days prior to overdose, n = 6131		MOUD in 90 days prior to overdose, n = 320	
		**Average Adjusted Prediction** (95% CI), %	***P***	**Average Adjusted Prediction** (95% CI), %	***P***
**Overdose Type**					
	Prescription	3.2 (2.6 to 3.8)	--	55.8 (45.6 to 66.0)	--
	Heroin	6.8 (5.6 to 8.1)	< .001	53.5 (46.2 to 60.9)	.75
**Age (years),** at mean		3.6 (3.0 to 4.1)	< .001	54.4 (49.2 to 59.6)	.22
**Sex**					
	Male	5.1 (4.5 to 5.7)	--	54.6 (47.9 to 61.3)	--
	Female	3.9 (3.2 to 4.6)	.03	54.0 (45.0 to 62.9)	.92
**Race/Ethnicity**					
	White	4.8 (4.3 to 5.3)	--	53.2 (47.1 to 59.3)	--
	Black	3.0 (1.6 to 4.4)	.03	71.8 (50.8 to 92.7)	.10
	Hispanic	4.1 (2.3 to 5.9)	.47	70.4 (50.8 to 90.0)	.10
	Asian	5.9 (-.19 to 12.1)	.71	62.0 (2.6 to 126.7)	.79
	Unknown	4.3 (2.6 to 6.0)	.60	40.5 (20.7 to 60.1)	.23
**Year**					
	2011 Q4	6.0 (2.9 to 9.2)	--	27.3 (-3.9 to 58.6)	--
	2012	4.2 (3.0 to 5.5)	.29	48.8 (34.6 to 63.0)	.22
	2013	6.4 (4.9 to 7.8)	.85	61.7 (50.4 to 72.9)	.05
	2014	3.4 (2.4 to 4.4)	.12	58.1 (45.1 to 71.0)	.07
	2015	4.4 (3.4 to 5.5)	.35	50.4 (37.8 to 63.1)	.18
	2016 Q1-3	4.4 (3.3 to 5.5)	.33	54.2 (42.6 to 65.9)	.11
**Region**					
	Northeast	4.4 (3.0 to 5.9)	--	55.3 (40.0 to 70.7)	--
	South	5.1 (4.2 to 6.0)	.46	51.9 (43.4 to 60.4)	.70
	Midwest	3.9 (3.0 to 4.8)	.52	58.7 (48.7 to 68.8)	.72
	West	4.8 (3.6 to 5.9)	.75	52.6 (40.0 to 65.4)	.79
**Anxiety treatment,** 90 d prior to overdose	No	4.3 (3.7 to 4.9)	--	54.5 (47.3 to 61.7)	--
	Yes	5.4 (4.2 to 6.6)	.13	54.1 (45.2 to 63.0)	.95
**Depression treatment,** 90 d prior to overdose	No	4.4 (3.8 to 5.0)	--	59.0 (52.7 to 65.3)	--
	Yes	5.0 (3.8 to 6.3)	.39	42.9 (32.5 to 53.2)	.01
**Prescription opioid claim,** 90 d prior to overdose	No	4.3 (3.6 to 4.9)	--	57.0 (50.4 to 63.4)	--
	Yes	5.1 (4.0 to 6.2)	.22	47.3 (35.2 to 59.3)	.20
**Benzodiazepine claim**, 90 d prior to overdose	No	4.0 (3.4 to 4.6)	--	53.9 (46.8 to 61.0)	--
	Yes	6.2 (2.9 to 7.5)	.003	55.0 (46.3 to 63.8)	.86
**Outpatient OUD Treatment,** 90 d prior to overdose	No	4.0 (3.5 to 4.5)	--	50.9 (42.6 to 59.1)	--
	Yes	10.1 (7.5 to 12.7)	< .001	57.2 (50.0 to 64.5)	.27

© 2020 Kilaru AS et al. *JAMA Network Open.*

eFigure 2
Kaplan-Meier Failure Curve for Days to First Follow Up Treatment Following Index ED Overdose[a]

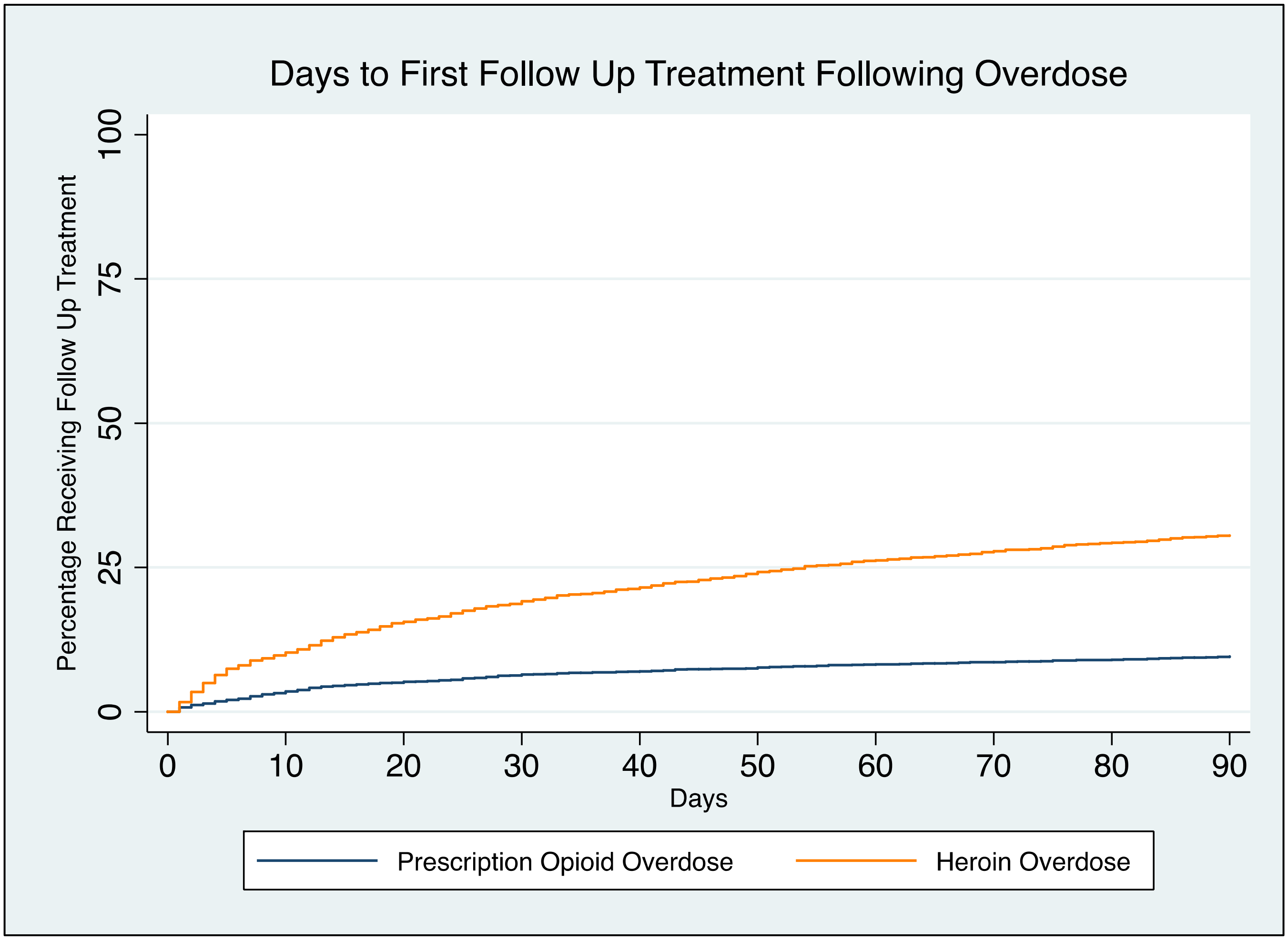

[a]Follow up treatment includes claim for OUD treatment encounter or pharmacy claim for MOUD

© 2020 Kilaru AS et al. *JAMA Network Open.*

eTable 6 Adjusted probability of follow-up treatment after opioid overdose, excluding patients without known claims beyond 90-day follow-up period (sensitivity analysis to address potential mortality during follow-up period).

		**No MOUD in 90 days prior to overdose,** n = 6131		**MOUD in 90 days prior to overdose,** n = 320	
		**Average Adjusted Prediction** (95% CI), %	***P***	**Average Adjusted Prediction** (95% CI), %	***P***
**Overdose Type**					
	Prescription	8.5 (7.5 to 9.4)	--	64.2 (57.5 to 70.1)	--
	Heroin	18.0 (15.8 to 20.2)	< .001	62.2 (56.8 to 67.6)	.69
**Age (years),** mean (SD)		10.1 (9.3 to 11.0)	< .001	63.1 (59.5 to 66.7)	.22
**Sex**					
	Male	12.4 (11.3 to 13.7)	--	62.5 (57.7 to 67.3)	--
	Female	10.4 (9.2 to 11.5)	.01	63.7 (58.2 to 69.1)	.76
**Race/Ethnicity**					
	White	12.5 (11.6 to 13.4)	--	63.3 (59.2 to 67.4)	--
	Black	6.3 (4.1 to 8.6)	< 0.001	75.8 (63.2 to 88.4)	.06
	Hispanic	9.1 (6.8 to 11.4)	.02	60.8 (46.6 to 74.9)	.73
	Asian	11.1 (3.2 to 19.1)	.75	60.0 (14.4 to 105.6)	.89
	Unknown	10.3 (7.6 to 13.1)	.15	53.0 (40.1 to 65.9)	.14
**Year**					
	2011 Q4	12.4 (8.0 to 16.7)	--	48.2 (22.0 to 74.2)	--
	2012	9.5 (7.7 to 11.4)	.24	66.2 (56.3 to 76.0)	.21
	2013	11.7 (9.7 to 13.6)	.77	66.6 (58.6 to 74.7)	.19
	2014	10.2 (8.4 to 11.9)	.36	61.0 (52.9 to 69.1)	.36
	2015	13.5 (11.7 to 15.4)	.62	56.7 (48.7 to 64.7)	.54
	2016 Q1-3	11.9 (10.1 to 13.8)	.86	67.0 (59.7 to 74.2)	.17
**Region**					
	Northeast	14.3 (11.9 to 16.8)	--	73.2 (64.5 to 81.8)	--
	South	10.7 (9.5 to 11.8)	.02	58.9 (52.3 to 65.4)	.01
	Midwest	11.4 (9.9 to 13.0)	.06	68.3 (62.0 to 74.7)	.37
	West	11.6 (9.8 to 13.4)	.09	53.5 (44.6 to 62.3)	.002
**Anxiety treatment,** 90 d prior to overdose	No	10.7 (9.8 to 11.6)	--	63.1 (58.2 to 67.9)	--
	Yes	14.0 (11.9 to 16.1)	.01	63.0 (57.1 to 68.9)	.98
**Depression treatment,** 90 d prior to overdose	No	11.4 (10.5 to 12.3)	--	63.9 (59.6 to 68.3)	--
	Yes	11.8 (9.9 to 13.8)	.70	61.0 (57.0 to 68.9)	.49
**Prescription opioid claim,** 90 d prior to overdose	No	11.5 (10.4 to 12.7)	--	65.8 (61.0 to 70.6)	--
	Yes	11.4 (10.0 to 12.9)	.93	57.3 (49.4 to 65.3)	.11
**Benzodiazepine claim**, 90 d prior to overdose	No	10.8 (9.8 to 11.7)	--	60.5 (55.8 to 65.1)	--
	Yes	13.3 (11.5 to 15.1)	.02	67.4 (61.4 to 73.4)	.08

© 2020 Kilaru AS et al. *JAMA Network Open.*

eTable7 Index Opioid Overdoses by specific ICD-9 or ICD-10 Diagnosis Code, with number and frequency for each diagnosis code

Diagnosis Code	Definition	Number	Frequency (%)
**965.00**	**Poisoning by opium (unspecified)**	**1135**	**17.59**
**965.01**	**Poisoning by heroin**	**1209**	**18.74**
965.02	Poisoning by methadone	139	2.15
**965.09**	**Poisoning by other opiates and related narcotics**	**2243**	**34.77**
E.850.0	Accidental poisoning by heroin	4	0.06
E.850.1	Accidental poisoning by methadone	2	0.03
E.850.2	Accidental poisoning by other opiates and related narcotics	21	0.33
ICD-10 [Starting October 1 2015]			
T400X1A	Poisoning by opium, accidental (unintentional), initial encounter	28	0.43
T400X2A	Poisoning by opium, intentional self-harm, initial encounter	2	0.03
T400X4A	Poisoning by opium, undetermined, initial encounter	3	0.05
**T401X1A**	**Poisoning by heroin, accidental (unintentional), initial encounter**	**535**	**8.29**
T401X2A	Poisoning by heroin, intentional self-harm, initial encounter	38	0.59
T401X4A	Poisoning by heroin, undetermined, initial encounter	51	0.79
**T402X1A**	**Poisoning by other opioids, accidental (unintentional), initial encounter**	**579**	**8.98**
T402X1D	Poisoning by other opioids, accidental (unintentional), subseequent encounter	2	0.03
T402X1S	Poisoning by other opioids, accidental (unintentional), sequelae	1	0.02
T402X2A	Poisoning by other opioids, intentional self-harm, initial enounter	160	2.48
T402X4A	Poisoning by other opioids, undetermined, initial encounter	62	0.96
T403X1A	Poisoning by methadone, accidental (unintentional), initial encounter	40	0.62
T403X2A	Poisoning by methadone, intentional self-harm, initial encounter	6	0.09
T403X4A	Poisoning by methadone, undetermined	1	0.02
T404X1A	Poisoning by other synthetic narcotics, accidental (unintentional), initial encounter	119	1.84
T404X2A	Poisoning by other synthetic narcotics, intentional self-harm, initial encounter	51	0.79
T404X4A	Poisoning by other synthetic narcotics, undetermined, initial encounter	20	0.31
**TOTAL**		6451	100.00

© 2020 Kilaru AS et al. *JAMA Network Open.*

eTable 8 Patient cohort and unadjusted outcomes, stratified by overdose type and treatment status before overdose

Index Emergency Department Opioid Overdose
n = 6451

Heroin Overdose
1896 (29.4%)

Prescription Opioid Overdose
4555 (70.6%)

	Heroin Overdose: **No MOUD or Treatment,** 90d prior to overdose 1476 (22.9%)	Heroin Overdose: **MOUD or Treatment,** 90d prior to overdose 420 (6.5%)	Prescription Opioid Overdose: **No MOUD or Treatment,** 90d prior to overdose 4293 (66.5%)	Prescription Opioid Overdose: **MOUD or Treatment,** 90d prior to overdose 262 (4.1%)
**Both MOUD and Treatment Encounter**, 90d after index opioid overdose	64 (4.3%)	101 (24.0%)	46 (1.1%)	38 (14.5%)
On**ly MOUD Claim,** 90d after index opioid overdose	51 (3.5%)	52 (12.4%)	62 (1.4%)	40 (15.3%)
**Only Treatment Encounter,** 90d after index opioid overdose	211 (14.3%)	121 (28.8%)	209 (4.9%)	74 (28.2%)
**No MOUD or Treatment Encounter,** 90d after index opioid overdose	1150 (77.9%)	146 (34.8%)	3976 (92.6%)	110 (42.0%)

© 2020 Kilaru AS et al. *JAMA Network Open.*
